# Prevalence of Colistin-resistant Gram-negative Isolates Carrying the *mcr-1 Gene* among Patients Visiting a Tertiary Care Center

**DOI:** 10.31729/jnma.5246

**Published:** 2020-12-31

**Authors:** Ashmita Paudel, Surya Prasad Devkota, Anima Shrestha, Anil Kumar Shah

**Affiliations:** 1 Department of Microbiology, Regional College of Health Science and Technology, Pokhara, Nepal; 2 Department of Microbiology, Pokhara Bigyan Tatha Prabidhi Campus, Pokhara, Nepal; 3 Department of Microbiology, Saint Xavier's College, Maitighar, Kathmandu, Nepal; 4 Annapurna Research Center, Maitighar, Kathmandu, Nepal

**Keywords:** *colistin-resistant*, *gram-negative isolates*, *mcr-1 gene*, *Nepal*

## Abstract

**Introduction:**

Gram-negative isolates harboring mobilized colistin resistance (*mcr-1*) gene are a great threat to human health. They have been reported worldwide among various bacterial isolates. This work aimed to study the prevalence of colistin resistance among Gram-negative bacteria and the incidence of *mcr-1* gene among these isolates.

**Methods:**

A descriptive cross-sectional study was done at a tertiary care center from June 2016 to February 2017. An ethical approval was taken from review board of the Nepal Health Research Council (Reg. no: 274/2016). Convenience sampling was used. The data was collected and analyzed using Microsoft Excel 2010 and Statistical Package for Social Sciences (SPSS) Version 16 . Point estimate at 95% Confidence Interval was calculated along with frequency and proportion for binary data.

**Results:**

Among 485 gram-negative isolates, only 13 (2.68%) (1.26-6.62 at 95% Confidence Interval) isolates were colistin-resistant and *mcr-1* was present in two isolates. Predominant colistin-resistant isolates were *E. coli* 6 (4.1%), *Enterobacter* spp 2 (2.81%), and *Acinetobacter* spp 2 (2.81%). A high level of colistin-resistance was noted in 4 (30.7%) isolates as indicated by the very high value of colistin MIC (>256 μg/ml). ICU was the major site of isolation of colistin-resistant and *mcr-1* positive pathogens. The majority of colistin-resistant isolates were highly drug-resistant and were sensitive only to polymyxin B. Antibiotics like imipenem, amikacin, gentamicin, aztreonam, ciprofloxacin, and piperacillin-tazobactam were effective for few of these isolates.

**Conclusions:**

Though the prevalence of *mcr-1* gene was low among colistin-resistant gram-negative isolates, the resistant pattern was quite alarming as these isolates were highly drug-resistant.

## INTRODUCTION

Colistin, although not used in routine treatment procedure due to its toxicity, is a very effective treatment option against the majority of multi-drug-resistant gram-negative pathogens.^1^ Colistin is active against isolates producing New Delhi Metallo-(β-lactamase and Klebsiella pneumoniae carbapenemase.^2^ The emergence of resistance to colistin among gram-negative pathogens is creating infections with very limited treatment options.^3^

A recently discovered enzyme, phosphoethanolamine transferase, encoded by the *mcr-1* gene is responsible for plasmid-mediated colistin resistance.^4^ This novel mechanism of resistance is a matter of concern as it may cause pan-drug resistance among the member of Enterobacteriaceae.^5^ At least six members of Enterobacteriaceae have been reported as the recipient of the highly diverse *mcr-1* bearing plasmids having complex dissemination mechanisms.^6^

This study was done to know the prevalence of colistin- resistant as well as *mcr-1* producing Gram-negative isolates as colistin resistance is increasing among Gram-negative isolates globally.

## METHODS

This descriptive cross-sectional study was done at Annapurna Neurological Hospital and Allied Sciences, Maitighar, Kathmandu, Nepal, and the study duration was June 2016 to February 2017. The research protocol was approved by an ethical review board of the Nepal Health Research Council (Reg. no: 274/2016). All patients attending the hospital with suspected bacterial infections were included in this study as a study population. Samples that were collected from patients currently under antibiotics therapy, samples with a clear sign of contamination, and those collected in improper containers were excluded from the study. The convenience sampling method was used and all the samples obtained during the study period were analyzed. The sample size (n) was calculated as,

$$\begin{array}{ll} \text{n} & \text{= }{\text{Z}}^{\text{2}}\times \text{p}\times \text{1}-\text{p}/{\text{e}}^{\text{2}}\\ & \text{= }{\left(1.96\right)}^{\text{2}}\times 0.0316\times 0.9684/{(\text{0.02)}}^{\text{2}}\\ & \text{= }293.89\\ & \approx 294 \end{array}$$

Where,

n = required sample size
Z = 1.96 at 95% Confidence Interval (CI)
p = prevalence of colistin resistance, 3.16%^7^
e = margin of error, 2%

Hence, the calculated sample size was 294 but we analyzed 485 isolates in our study.

So, 485 Gram-negative bacteria from various clinical specimens were included in the study excluding intrinsic colistin-resistant Gram-negative bacteria as reported earlier.^3^ The identification of isolates was done by routine biochemical tests and colony characteristics. Isolates resistant to colistin using discs diffusion (10μg, Himedia-Laboratories, India), as per CLSI guidelines^8^ were further examined by colistin MIC detection using E-stripes to confirm phenotypic colistin-resistance. E. coli ATCC 25922 was used as a negative control. Isolates resistant to colistin were selected for further study and preserved using glycerol stock until plasmid extraction.

**Figure 1:**
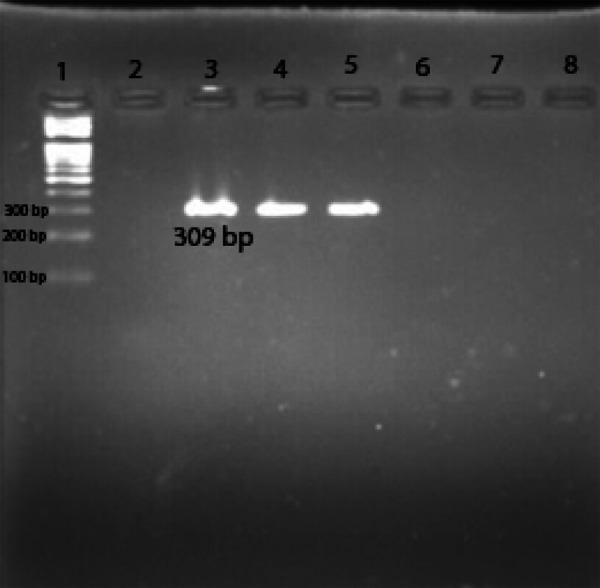
PCR amplified *mcr-1* gene on agarose gel.

For the detection of a 309 bp internal fragment of mcr-1 gene, 5μl of the plasmid DNA was subjected to PCR using specific primers; mcr-1 Forward (5'-CGGTCAGTCCGTTTGTTC-3') and *mcr-1* Reverse (5'-CTTGGTCGGTCTGTAGGG-3') as reported already.^4,9^ The positive control used was mcr-1 positive plasmid of E.coli while the plasmid of E. coli ATCC 25922 was used as a negative control. The thermal cycling process reported by Cavaco et al^9^ using *mcr-1* specific primers was followed for PCR. Amplified products were visualized after electrophoresis on the agarose gel. DNA ladder (100 bp) was used as a molecular weight marker where an amplified product having size 309 bp was regarded as *mcr-1* positive (Figure 1).

Lane1-100bp DNA ladder, Lane 2-blank, Lane 3-mcr-1 positive control (309bp), Lane 4-5 mcr-1 positive, Lane 6-8 mcr-1 negative

Data entry and analysis were done in the Microsoft Excel 2010 and Statistical Package for the Social Sciences version 16. Descriptive statistics were expressed as frequency and proportion for binary data.

## RESULTS

A total of 485 gram-negative isolates were included in the study. Among these isolates, only 13 (2.68%) (1.26-6.62 at 95% CI) were resistant to colistin by phenotypic methods. Colistin resistance was higher in E.coli 6 (4.10%) isolates followed by Enterobacter spp 2 (2.81%) and Acinetobacter spp 2 (2.81%). The majority i.e 8 (61.5%) of colistin-resistant isolates were from the Intensive care Unit (ICU) followed by an emergency ward, surgery ward, and outpatient department (OPD) (Table 1).

**Table 1:** Characteristics of colistin-resistant Gram-negative isolates.

Isolate	Specimen	Ward	Colistin MIC μg/ml	AST profile		*mcr-1* gene
				Resistant	Sensitive	
E.coli	Urine	OPD	>256	^††^CAZ, ^‡‡^CTR, ^†^PB, ^*^IPM, ^‡^AT, ^**^CIP, ^||^AK, ^¶^GEN	PIT	Negative
E.coli	Urine	ICU	4	CAZ,CTR,AT,AK,IPM, ^§^PIT,CIP	PB, GEN	Negative
E.coli	Pus	Surgery	4	CAZ,CTR,CIP,AT,PIT,AK,GEN	PB, IPM	Negative
E.coli	Urine	ICU	96	CAZ,CTR,IPM,CIP,AK,GEN,PIT	PB,AT	Positive
E.coli	Sputum	ICU	8	CAZ,CTR,IPM,CIP,AK,GEN,PB,AT	PIT	Negative
Klebsiella oxytoca	Urine	Emergency	6	CAZ,CTR,IPM,CIP,PIT,AK,GEN	PB,AT	Negative
Klebsiella pneumonia	Blood	ICU	>256	CAZ,CTR,PB,IPM,AT,CIP,PIT,GEN	AK	Positive
Klebsiella pneumonia	Sputum	Emergency	>256	CAZ,CTR,IPM,AT,CIP,GEN,PIT	PB,AK	Negative
Acinetobacter spp	Urine	ICU	32	CAZ,CTR,PB,AT,CIP,AK,GEN	PIT,IPM	Negative
Acinetobacter spp	Sputum	ICU	6	CAZ,CTR,IPM,CIP,AK,GEN,PIT	PB,AT	Negative
Pseudomonas spp	Blood	ICU	4	CAZ,CTR,PB,IPM,AT,CIP,AK	PIT,CIP	Negative
Enterobacter spp	Pus	Surgery	8	CAZ,CTR,IPM,AT,CIP,AK,PIT	PB,GEN	Negative
Citrobacter spp	Body fluid	ICU	>256	CAZ,CTR,PB,AT,CIP,PIT,IPM,GEN	AK	Negative

* IPM=Imipenem,

† PB=PolymyxinB,

‡ AT=Azteronam,

§ PIT=Piperacillin-Tazobactam,

|| AK=Amikacin,

¶ GEN=Gentamicin,

** CIP=Ciprofloxacin,

†† CAZ=Ceftazidime,

‡‡ CTR=Ceftriaxone

The range of colistin MIC for these isolates was 4 to >256 μg/ml (Table 1). Colistin-resistant isolates were highly drug-resistant, as all of these pathogens were resistant to ceftazidime and ceftriaxone and resistance was high for antibiotics like imipenem, ciprofloxacin, gentamicin, amikacin, aztreonam, and piperacillin- tazobactam. The highest rate of sensitivity was noted only to polymyxin B as 7 isolates (54%) were sensitive to it indicating this antibiotic as a treatment option for the majority of these pathogens.

Out of the 13 colistin-resistant isolates, only two (15.38%) isolates were positive for *mcr-1* gene with the *mcr-1* gene found in 0.41% of total Gram-negative isolates. One (0.68%) isolate of *E.coli* of all *E.coli* isolates and one (1.04%) *Klebsiella pneumoniae* of all Klebsiella isolates were positive for the gene. Although colistin-resistant isolates were from different clinical samples, the *mcr-1* gene was detected only from urine and blood isolate. *mcr-1* positive isolates were obtained from both male and female patient one each having age group 15-45 years and 45 years above respectively. *mcr-1* positive isolates were extremely drug-resistant as they were resistant to almost all routine antibiotics. mcr-1 positive *Klebsiella pneumoniae* isolate was resistant even to polymyxin B while the other was sensitive to it.

## DISCUSSION

The prevalence of colistin-resistant Gram-negative isolates found in this study was comparable to the study by Wong et al.^10^ Similarly, another study has reported that less than 10% isolates were resistant to colistin globally whereas the pattern was quite higher in India and the Philippines with 13.8% and 50% prevalence respectively.^11^ This variation may be the result of geographic variation, the difference of study period, the methodology used, and variation in colistin use, etc.

The distribution of colistin resistance among gram-negative isolates within hospital wards presents a horrible picture as 8 out of 13 colistin-resistant isolates were from intensive care unit patients. Various factors may be contributing to the emergence of these isolates in inpatients are prolonged stay in ICU, the process of surgery, use of colistin as well as third-generation cephalosporins and monobactams as indicated by Matthaiou's study^12^ and either inadequate or excess use of colistin.^13^ The high incidence of these isolates in the ICU and other wards poses a great challenge for their treatment. Not only this, these wards may act as a silent active source of dissemination of these highly notorious pathogens to other patients.

In this study, we reported variable colistin MIC values of the colistin-resistant isolate. These findings were in accordance with the finding of Liassine et al where they have reported the colistin MIC range of 4 to >128 mg/l.5 Similarly, various other studies have reported highly variable colistin MIC values of such isolates.^10,11,13^ This increasing resistance against colistin may be due to increased use of colistin for the treatment of MDR gram-negative isolates by clinicians.^11^

Isolates that were resistant to a last-line drug, colistin, were also resistant to many of the routine antibiotics. Such a high drug resistance among such pathogens has been reported by various authors.^3,4,13^ However, they were not resistant to all available antibiotics as found in this study because Polymyxin B was effective against the majority of these isolates and other antibiotics like imipenem, amikacin, gentamicin, aztreonam, and ciprofloxacin were effective against some of the isolates. As reported by other researches, treatment options for colistin-resistant isolates may be combination therapy.^3^ These results indicate that some of these isolates are still treatable by various classes of antibiotics as indicated by Walkty et al.^2^

The incidence of mcr-1 gene among colistin-resistant isolate was 15.3%, which was very analogous to the findings of Walkty et al.^2^ In this study, less than 0.5% gram-negative isolates (excluding intrinsic colistin resistant spp) were positive for *mcr-1* gene. The study of Fernandes et al also reported similar results.^14^ In addition to these, a very matching prevalence of mcr-1 gene among enterobacterial isolates was reported by Wong et al.^10^ This low prevalence of the plasmid-mediated *mcr-1* gene indicates that there is another significant mechanism for colistin resistance among gram-negative isolates in this region and may be due to chromosomal or by other mcr variants reported by Zhang et al.^15^ There is an urgent need to detect such mechanisms for the proper control and management of these superbugs.

Out of different colistin-resistant pathogens, only *E.coli* and *Klebsiella pneumoniae* harbored the gene with a prevalence of around 1%. Similarly, Quan et al reported that 1.1% *E.coli* isolates (20 out of 1495) and <1% *Klebsiella* spp (1 out of 571) were *mcr-1* positive.^16^ The presence of mcr-1 gene mostly in *E.coli* and/or *Klebsiella* spp is supported by other studies as well.^10,17,18^ Colistin-resistant isolates from urine and blood were positive for the gene. These findings are comparable with the findings of Wong et al.^10^ Similarly, the prevalence of colistin resistance was higher in urine, sputum and blood isolate in our study as like the findings of Arjun et al. where the blood, urine, and respiratory isolates were predominant colistin-resistant isolates.^3^

The emergence of gram-negative isolates resistant to last-line drugs like colistin and polymyxin B is a matter of concern as some of the isolates in this study were extremely drug-resistant having very limited treatment options. Likewise, colistin resistance has been reported from Canada,^2^ India,^3^ South Africa^4^, Greece,^13^ and Australia^19^ as well. These isolates are limited not only in *E.coli* and *Klebsiella* spp, but they also have been detected in *Acinetobacter, Pseudomonas, Enterobacter, Citrobacter, Salmonella, Pantoea^20^* and various other gram-negative isolates. This global dissemination of these pathogens among the majority of significant human pathogens may mimic the rapid spread as that of New Delhi Metallo-β-lactamase and poses a possibility of reverting the situation to the preantibiotic era in near future.

This study has some limitations as this study was focused only on colistin-resistant isolates to detect *mcr-1* gene but there is a case of detection of this gene in colistin susceptible isolates.^1^ Moreover, only clinical isolates were taken into consideration while there are several reports of *mcr-1*detection from food, food animals, and environmental isolates.^21^ Screening of clinical, food, and various environmental samples to detect these pathogens as well as detail molecular analysis is necessary to get the real picture of this problem in this region.

## CONCLUSIONS

This study has detected the plasmid-mediated colistin-resistant gene *mcr-1* in Nepal for the first time among clinical gram-negative isolates. Less than 3 percent of gram-negative isolates were found to be colistin- resistant. The incidence of *mcr-1* gene was low among these isolates. *E.coli* and *Klebsiella* were found to harbor *mcr-1* gene. Colistin-resistant isolates were more prevalent in ICU increasing the treatment difficulty of vulnerable patients. colistin-resistant isolates were highly drug-resistant and were not susceptible to almost all routine antibiotics.
